# Modelling Stream-Fish Functional Traits in Reference Conditions: Regional and Local Environmental Correlates

**DOI:** 10.1371/journal.pone.0045787

**Published:** 2012-09-24

**Authors:** João M. Oliveira, Pedro Segurado, José M. Santos, Amílcar Teixeira, Maria T. Ferreira, Rui V. Cortes

**Affiliations:** 1 Centro de Investigação e de Tecnologias Agro-Ambientais e Biológicas (CITAB), Universidade de Trás-os-Montes e Alto Douro, Vila Real, Portugal; 2 Centro de Estudos Florestais (CEF), Instituto Superior de Agronomia, Universidade Técnica de Lisboa, Lisboa, Portugal; 3 Centro de Investigação de Montanha (CIMO), Escola Superior Agrária, Instituto Politécnico de Bragança, Bragança, Portugal; Swansea University, United Kingdom

## Abstract

Identifying the environmental gradients that control the functional structure of biological assemblages in reference conditions is fundamental to help river management and predict the consequences of anthropogenic stressors. Fish metrics (density of ecological guilds, and species richness) from 117 least disturbed stream reaches in several western Iberia river basins were modelled with generalized linear models in order to investigate the importance of regional- and local-scale abiotic gradients to variation in functional structure of fish assemblages. Functional patterns were primarily associated with regional features, such as catchment elevation and slope, rainfall, and drainage area. Spatial variations of fish guilds were thus associated with broad geographic gradients, showing (1) pronounced latitudinal patterns, affected mainly by climatic factors and topography, or (2) at the basin level, strong upstream-downstream patterns related to stream position in the longitudinal gradient. Maximum native species richness was observed in midsize streams in accordance with the river continuum concept. The findings of our study emphasized the need to use a multi-scale approach in order to fully assess the factors that govern the functional organization of biotic assemblages in ‘natural’ streams, as well as to improve biomonitoring and restoration of fluvial ecosystems.

## Introduction

Biomonitoring and stream restoration are important tools to re-establish the health of river ecosystems with the emphasis placed on the restoration of ecological processes and ecosystem functionality [1]. Therefore, identifying the primary ‘natural’ environmental gradients that control biological assemblages at the functional level should provide information to help river management and predict the consequences of anthropogenic stressors.

The structure of any local biological assemblage is a function of several biotic and abiotic factors operating on multiple spatial and temporal scales [2], [3]. In fact, many studies (e.g., [4]–[10]) have shown that both local factors, such as channel morphology and fish cover, and regional factors (many of which are correlated with one another and influence local conditions), such as climate, and both large-scale land use and water transfer, can contribute to the structure of fish assemblages. The question of which spatial scale reveals the strongest relationships among environmental variables and fish assemblages has attracted considerable interest, although those conclusions may depend on the biological unit employed (species/functional groups) [8], the degree of anthropogenic landscape disturbance [7], [11], or the geo-climatic heterogeneity of the studied area [9]. Of the studies that directly addressed this question in least disturbed regions, Wang et al. [7] reported stronger relations at a local scale in north-central United States, and Esselman and Allan [9] for Mesoamerican streams suggested that landscape-scale factors had stronger relative influence on fish assemblages. In Europe, Ferreira et al. [12] for Western Iberia and Pont et al. [13] for France reported that both watershed and reach-scale factors were equally important in structuring fish species. Although all these studies have contributed greatly to our understanding of fish-environment relations across multiple spatial scales at the taxonomic level, little attention has been paid to the factors that determine the functional organization of stream-fish assemblages in reference conditions (but see [7]). Recently, Logez et al. [14] tested the functional structures of some European fish assemblages in least disturbed conditions, but they only used five environmental variables. We are unaware of any studies in European undegraded streams evaluating the roles of a large set of local and regional environmental factors in determining the functional organization of fish assemblages.

The functional perspective has been recently emphasized in stream-fish ecology, in which the assemblage traits are the primary focus [8], [15]–[17]. This can be achieved by distinguishing functional groups (or guilds): groups of species that exploit the same class of environmental resources in a similar way [18]. Species can be grouped into guilds on the basis of many different life-history traits (e.g., reproduction, feeding, habitat), reflecting morphological, physiological, and behavioural adaptations to their environment. Thus, species traits can be used to examine relations between the fundamental ecological function of fish assemblages and the environmental variables in natural conditions [19], [20]. The functional guild approach is the cornerstone for the development of multi-metric indices, such as the Indices of Biotic Integrity, to assess the biological condition of aquatic systems across large landscapes [21]–[22]. The use of guilds has several advantages: (1) species in a guild are more likely than an entire assemblage or taxonomic group to respond to a common subset of environmental variables [23]; (2) density of individual species may be more fluctuating via biotic and abiotic factors than density of whole guilds [24]; (3) since the use of the guild concept is largely less geographically constrained than the taxonomic level, it may be more useful for comparing the organization of fish assemblages across large geographic scales [25].

In riverine systems fish assemblages may reveal significant variations along broad geographic gradients, suggesting adaptation to spatial (i.e. latitudinal and longitudinal) changes in abiotic variables. For example, Marsh-Matthews and Matthews [26], in a study of streams from Iowa to Texas, reported that variations in fish assemblage composition reflected the overall north-south arrangement of drainages sampled. Ferreira et al. [27] described latitudinal variations of Portuguese fish assemblages, based on climatic and topographic factors, and Pusey and Kennard [28] also found a strong north-south gradient in structure of fish assemblages of Northern Queensland. Several works have addressed the phenomenon of longitudinal variation of both fish species richness and fish functional groups (e.g., [9], [15], [25], [29]–[33]). These studies have demonstrated that variables that represent longitudinal position in the catchment, including local habitat characteristics (e.g., depth, width, slope, discharge) and its regional drivers (e.g., climatic variables, drainage area) correlate strongly to fish assemblage variation. For example, McGarvey and Hughes [32] and McGarvey and Ward [33] described longitudinal patterns in both fish guilds and species richness as a function of river discharge across large geographic areas. These patterns are usually attributed to concepts that relate the gradient in physical factors that occur along river systems, to changes in assemblage structure and function.

In this study we use a combination of multivariate statistical techniques to further our objectives: (1) to identify the regional- and local-scale environmental gradients explaining the functional structure of western Iberian fish assemblages at least disturbed sites (reference conditions); and (2) to assess the relative importance of these abiotic gradients on the abundance of fish guilds and fish species richness. We also hypothesized that the generic biological patterns would be associated with broad geographic gradients, with variations of the functional organization of fish assemblages either with latitude or along the streams.

## Methods

### Ethics Statement

All animal work (from sampling to handling) was conducted in accordance to relevant national and international guidelines to minimise discomfort to individuals [34], [35]. The necessary permits for fish sampling were obtained from the National Forest Authority.

### Study Area

**Figure 1:**
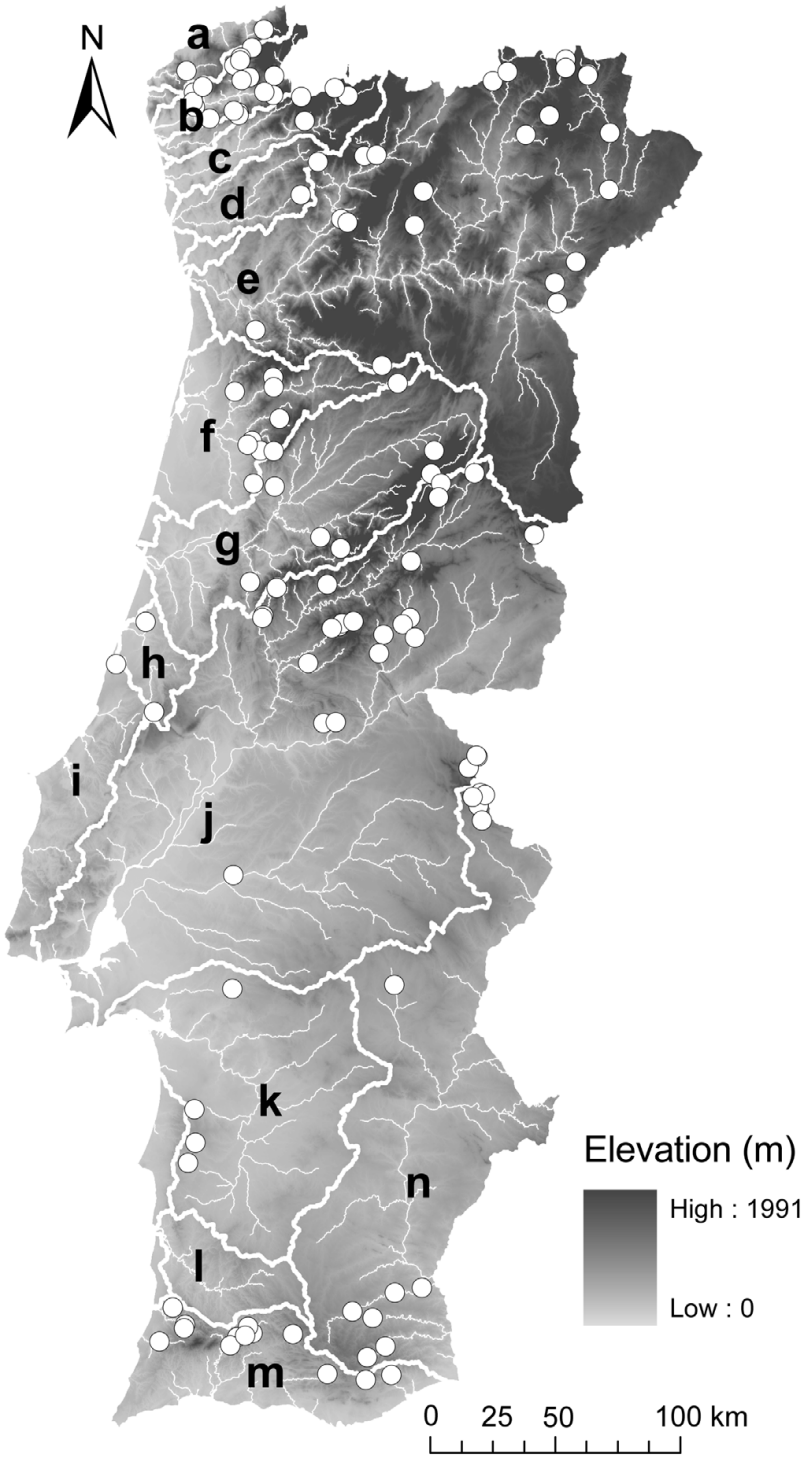
Location of the sampling sites. The elevation and the main river basins are also shown (a - Minho; b - Lima; c - Cávado; d - Ave; e - Douro; f - Vouga; g - Mondego; h - Lis; i - Extremadura coastal basins; j - Tagus; k - Sado; l - Mira; m - Algarve costal basins; n - Guadiana).

The study area is located on the west side of the Iberian Peninsula and includes all the hydrological network of Portuguese catchments (Figure 1), with most of the rivers running in a NE–SW direction towards the Atlantic coast. Geology of the country is complex. The inland area is dominated by the pre-Mesozoic Hesperic Massif – a geological unit that includes granites, schists and quartzites with various degrees of metamorphism – whereas the coast is dominated by tertiary layers under quaternary deposits, but including a few calcareous areas; in the southern region, below Tagus river, the flat platforms of the Hesperic Massif predominate in extensive areas and further south, geology is mainly composed of sedimentary deposits (e.g., sandstones, limestones, conglomerates and marls), mainly with a calcareous background.

The climate reflects both the elevation and the proximity of the Atlantic Ocean, ranging from temperate oceanic on the northwest of the study area, to Mediterranean in most of the country [36]. Temperatures increase southwards and mean annual precipitation is generally higher in the northern area. The rainfall pattern exhibits strong intra-and inter-annual variability, with a discharge regime that is typical of Mediterranean systems [37]. Therefore, high floods occur from autumn to late-winter, with a gradual decline in flow that eventually dries out during late spring and summer in the most arid areas.

The landscape varies greatly across the study area. The northern and central part of the country part is dominated by narrow and steep valleys, mostly forested with English oak (*Quercus robur*) and Pyrenean oak (*Quercus pyrenaica*). Riparian forests are dense and typically dominated by the common alder *(Alnus glutinosa)* and the narrow-leafed ash (*Fraxinus angustifolia*), frequently found in association with willows (*Salix* spp.) and poplars (*Populus* spp.). Pines *(Pinus* spp.*)* and *Eucalyptus* plantations are also characteristic of this area, which also supports a patchy pattern of orchards, vineyards, olive groves and irrigated crops. The southern area is more homogenous and largely characterized by agriculture, Mediterranean shrublands and evergreen oak (*Quercus suber* and *Quercus ilex*) woodlands. Meander valleys and small floodplains are characteristic of the middle and lower reaches of the main rivers. Most streams in this region have narrow riparian forests, primarily dominated by willows and ashes, which, in the driest areas, are replaced by shrublands mainly composed by the oleander (*Nerium oleander*) and the Ibero-African shrubby spurge (*Flueggea tinctoria*).

The biogeographic isolation of the Iberian Peninsula by the Atlantic Ocean and the Pyrenees allowed the development of a characteristic fish fauna, similar to other European Mediterranean peninsulas [38]–[39], characterized by a low number of families, with most of the species belonging to the family Cyprinidae, a high degree of diversification at the species level, and the greatest European percentage of endemics [40]. There is also a long history of alien fish introductions representing a total of twenty three species, with more than half of the introductions occurring after the beginning of 20^th^ century, mainly for fishery and ornamental purposes [41].

### Site Selection

We selected data from fish samples of Portuguese streams collected by a consortium of Portuguese national universities 1996–2006. From a collection of more than 500 sites, only least disturbed sites were considered (‘reference data set’ or RD). The identification followed the EU-FAME project approach [16], [42], in which human disturbance is ranked semi-quantitatively using all available field data and GIS information. In our study we considered fifteen disturbance variables at catchment, segment and reach scales (Table S1), that were scored to the degree they deviated from minimally disturbed conditions (from 1 for no deviation, to 5 for highly degraded). The RD included sites with a classification of 1 and 2 on, at least, 80% of the 15 variables, allowing a maximum classification of 3 on 20% of those variables, with the exception of the ‘alien fish abundance’, which must be included in class 1 (no aliens) or 2 (<15% aliens). Thus, a median pressure classification of three was allowed for no more than three abiotic variables. This allowance was based on the assumption that minor deviations from excellent/good conditions do not significantly alter biological patterns at the assemblage level [27]. In addition, only sites spaced out 2 km from each other were retained for analyses to reduce problems of spatial autocorrelation while maintaining a sufficiently large sample size [43]. This yielded a total of 117 stream reaches (mean stream width = 5.6 m; min 1.0 m-max 30.0 m) from all main Portuguese catchments (Minho, Lima, Cávado, Ave, Douro, Vouga, Mondego, Lis, Extremadura coastal basins, Tagus, Sado, Guadiana and Algarve costal basins) (Figure 1), that cover the range of natural conditions across the country (Table 1).

**Table 1:** Median and range of environmental variables measured at 117 least disturbed sites in Portugal.

Environmental variables		Median (range)
**Regional variables**	Catchment area (km^2^)	38.00 (4.40–1340.00)
	Catchment shape index	1.08 (0.20–4.88)
	Stream order	2 (1–6)
	Catchment mean elevation (m)	599.00 (75.00–1256.00)
	Catchment elevation range (m)	612.00 (111.00–1421.00)
	Catchment mean slope (%)	0.57 (0.08–2.11)
	Catchment slope range (%)	1.75 (0.09–5.87)
	Mean flow accumulation	82.00 (21.00–487.00)
	Drainage density (km/km^2^)	0.94 (0.62–1.33)
	Mean annual precipitation (mm)	1107.00 (556.00–1612.00)
	Mean summer precipitation (mm)	25.00 (6.00–49.00)
	Summer ombrothermic index	2.10 (0.59–4.75)
**Local variables**	Elevation (m)	247.00 (6.00–952.00)
	Channel slope (%)	1.04 (0.01–16.40)
	Channel sinuosity	1.45 (1.11–2.22)
	Mean annual temperature (°C)	13.54 (8.81–16.97)
	Mean July temperature (°C)	21.60 (17.70–24.60)
	Conductivity (µS/cm)	63.00 (10.00–769.00)
	Water temperature (°C)	17.80 (7.80–29.80)
	Mean width (m)	5.60 (1.00–30.00)
	Mean depth (m)	0.40 (0.13–1.00)
	Maximum depth (m)	1.00 (0.20–3.00)
	Mean width-depth ratio	14.00 (2.86–70.67)
	Dominant substrate (class)	5 (2–7)
	Woody cover (class)	3 (1–5)
	Macrophyte cover (class)	2 (1–5)
	Shading (class)	3 (1–5)

### Fish Sampling

During spring–summer base flow, sites were electrofished (DC, 300–700 V, or pulsed DC, 400–1000 V) once. In this period stream flows were lower (but still had full connectivity between habitats), thus ensuring a higher fishing efficiency, while at the same time, we avoided the extreme-flow events that typically occur during the rainy season from causing bias in fish sampling or in the measurement of local habitat variables [43]. Electrofishing distances followed CEN standards for assessing fish species composition and abundance for a given site (point 3.3.2 in [34]). This distance was at least 20 times the mean wetted width of the channel to encompass complete sets of the characteristic stream form (e.g., riffles, pools, runs); minimum sampled length was 100 m, and maximum sampled length was 600 m. The entire widths of wadeable streams were fished by walking slowly upstream and using one anode for every 5 m of stream width. The majority of sites were sampled by wading, but a few rivers with mean depths exceeding 0.8 m were electrofished by boat moving downstream, again sampling all habitat types, but focusing on the margins. Although capture efficiency estimates, assumed to be constant across sites, were not available for the study area, previous works indicated that this sampling effort was sufficient to ensure accurate characterization of fish species composition and abundance in the studied streams [27], [43]–[44]. Fish were identified and measured in the field and returned alive to the water. Because juveniles of the four *Luciobarbus* species (*L. comizo*/*microcephalus*/*sclateri*/*steindachneri*) present in the southern Portuguese basins can not be reliably identified to species, we grouped them as *Luciobarbus* spp. For the same reason we grouped *Lampetra fluviatilis* and *L. planeri*.

### Fish Metrics

To test whether the functional structure of fish assemblages responded to environmental gradients, we considered ten metrics related to the total density of fish guilds grouped into five ecological functions, following Noble et al. [23]: 1) overall tolerance guilds, based on the ability of species to survive and reproduce in a wide range of natural environmental conditions (non-tolerant (NOTO), and tolerant (TOLE)); 2) trophic guilds, based on food items in the diet of adult individuals (invertivores (INVE) and omnivorous (OMNI)); 3) feeding habitat guilds, based on the preferred habitat to live and feed (benthic (BENT) and water column (WACO)); 4) reproduction guilds, based on spawning substrate (lithophilic (LITH), phytolithophilic (PHLI), and polyphilic (POLY)); 5) migratory behaviour guild (potamodromous species (POTA)). The fish species were generally assigned for these guilds based on Fame Consortium [45], [46] with a few modifications supported by more recent grey or published data, completed by expert judgment when necessary (Table 2). These assemblage traits represent important aspects of the biology and ecology of fish species [14], [23], and could be considered of great interest to environmental assessment and management [19]. Following Logez et al. [14], we also considered number of native species (NATI) and total density of native individuals (DENS) as metrics.

**Table 2:** Catch (median and 1st-3rd quartiles; individuals per 1000m^2^), frequency of occurrence (FO) (%), index of relative dominance (IRD), and functional guilds for fish taxonomic groups collected in Portugal.

Species	Catch	FO	IRD	Guilds
*Acondrostoma oligolepis*	68 (32–106)	17.1	195.2	TOLE, OMNI, WACO, PHLI
*Anguilla anguilla*	10 (5–22)	30.8	75.1	TOLE, INVE, BENT
*Cobitis paludica*	7 (2–21)	24.8	42.7	TOLE, INVE, BENT, POLI
*Iberochondrostoma almacai*	13 (11–17)	4.3	1.2	NOTO, OMNI, PHLI
*Iberochondrostoma lemmingii*	11 (5–19)	10.3	8.5	NOTO, OMNI, PHLI
*Iberochondrostoma lusitanicum*	11 (7–15)	5.1	0.8	TOLE, OMNI, PHLI
*Luciobarbus bocagei*	20 (8–70)	29.1	226.0	TOLE, OMNI, BENT, LITH, POTA
*Luciobarbus* spp.	54 (7–199)	10.3	62.2	TOLE, OMNI, BENT, LITH, POTA
*Lampetra* spp.	21(16–104)	4.3	5.5	NOTO, BENT, LITH
*Pseudochondrostoma duriense*	21 (8–51)	26.5	114.1	NOTO, OMNI, BENT, LITH, POTA
*Pseudochondrostoma polylepis*	42 (30–67)	12.0	29.7	NOTO, OMNI, BENT, LITH, POTA
*Salmo trutta*	35 (17–81)	53.8	587.0	NOTO, INVE, WACO, LITH, POTA
*Squalius alburnoides*	93 (54–183)	32.5	525.8	NOTO, INVE, WACO, LITH
*Squalius aradensis*	157 (45–353)	9.4	91.0	NOTO, INVE, WACO, LITH
*Squalius carolitertii*	25 (9–53)	35.0	242.3	NOTO, INVE, WACO, LITH
*Squalius pyrenaicus*	70 (29–219)	32.5	634.1	NOTO, INVE, WACO, LITH
Aliens	9 (4–18)	13.0	0.7	

Only fish taxa presented at least at five sites are presented. The IRD for each group was calculated by multiplying the percent frequency of occurrence by the percent of relative abundance of that group. Guild abbreviations are defined in Methods.

### Environmental Data

For each site, environmental data were obtained at regional and local scales (Table 1). Following Hoeinghaus et al. [8], we considered regional factors as those associated with processes occurring over an area larger than the in-stream scale. Regional environmental variables included: (1) catchment area, catchment shape index (perimeter-area ratio), stream order, catchment mean elevation, catchment elevation range, catchment mean slope, catchment slope range, mean flow accumulation and drainage (stream) density (all these variables were partially derived from a Digital Terrain Model, with approximately 90 m resolution, from the NASA Shuttle Radar Topographic Mission, that is available from the CGIAR-CSI SRTM 90m Database: http://srtm.csi.cgiar.org/, accessed 2012 January 16); (2) mean annual precipitation, mean summer precipitation, and summer ombrothermic index [47] (these climatic variables were determined from 30 seconds (600–800 metres) resolution maps of monthly precipitation and monthly temperature that are available in the WorldClim website: http://www.worldclim.org/, accessed 2012 January 16). Regional variables were computed in a GIS environment using ArcGIS 9.2 (ESRI Inc., Redlands, CA, USA). Hydrology function from the ArcGIS Spatial Analyst package was used to produce the variables related to the river catchment network. Local environmental variables included: (1) elevation, channel slope, and channel sinuosity (stream length/valley length) (all derived from a Digital Terrain Model); (2) mean annual temperature and mean July temperature (both determined from the climate models described above); (3) water temperature and conductivity (both quantified at mid-channel, using a multiparameter meter); (4) dominant substrate (1 = silt, <0.02 cm; 2 = sand, 0.02–0.2 cm; 3 = gravel, 0.2–1.6 cm; 4 = pebble, 1.6–6.4 cm; 5 = cobble, 6.4–26.0 cm; 6 = boulder, >26.0 cm; 7 = bedrock), mean wetted width, mean depth, and maximum depth (all collected from 3 to 10 cross-sectional transects depending on distance fished (i.e. about one transect per 30 m of electrofishing distance)); (5) woody cover (woody debris and submerged roots), macrophyte cover, and overhanging tree shading (all visually assessed and scored on a grade scale for cover with the following classes (1 = <5%; 2 = 5–25%; 3 = 25–50%; 4 = 50–75%; 5 = >75%).

### Statistical Analyses

To reduce multicollinearity among environmental variables, principal component analysis (PCA), using Varimax rotation to ease interpretability [48], was performed separately on regional and local level variable groups. The reduced set of principal components, that still retained enough environmental variation, was used as the predictor gradients set in posterior analyses.

To investigate the relative influence of regional and local environmental gradients on the considered fish metrics, Generalized Linear Models (GLM; [49]) were adjusted. For functional guilds and DENS (density-based metrics), GLM were based on Negative Binomial error distribution with *log* as the link function. For NATI, GLM based on Poisson error distribution with *log* as the link function was used. The quadratic terms of all predictors were tested for inclusion in models, in order to account for unimodal responses of fish metrics.

Model selection procedure was based on the Information Theoretic Approach (ITA) [50], using the Akaike Information Criteria (AIC) as a measure of information loss of each candidate model, with the best fitting model having the lowest AIC. The difference between the AIC of each candidate model and the AIC of the top-ranked model was computed (Δi), in order to identify those models with larger support. Akaike weights (*wi*) were also computed for each candidate model, representing the probability of each model being selected as the best fitting model if the data were collected again under identical circumstances. Parameters of the final model were computed as the average of the parameters of models weighted by the respective *wi*. Only models with Δi ≤2, which are typically those considered to have substantial support, were considered in model averaging [50]. The relative importance of each variable was assessed using the probability of each variable to be included in the best approximating models. This was estimated by summing the *wi* of all candidate models where the variable was included.

All statistical analyses were performed using functions and routines implemented in R software version 2.12 [51].

## Results

### Fish Assemblages

We collected 29 species of fish from 9 families, representing a total of 17 822 individuals. About 82% of this total were endemic cyprinids. The aliens were represented in 15 sites (13%) and contributed only 1% of the total abundance (in eleven of these sites the relative abundance was less than 10%). Species richness ranged from 1 to 7 with a median of 3. The catch for the sampling sites ranged from 13 to 1623 and had a median of 179 individuals per 1000 m^2^. As expected, fish species with large distributions in Portugal were the most common (Table 2), mainly *Salmo trutta*, species from the genus *Squalius*, *Luciobarbus bocagei* and *Anguilla anguilla*. Conversely, most of the rare species in our study are endemic to small catchments or have restricted distribution in larger basins. Fish assemblages in the northern and central regions were dominated by *A. oligolepis*, *P. duriense*, *L. bocagei*, *S. carolitertii*, and *S. trutta* in the basins north of Tagus basin, and by *P. polylepis*, *L. bocagei*, *S. pyrenaicus*, and *S. alburnoides* in the Tagus basin. In the Guadiana basin three fish groups exhibited clear dominance: *Luciobarbus* spp., *C. paludica*, and *S. pyrenaicus*/*alburnoides*. The small cyprinid *S. aradensis* dominated the southern small Mediterranean-type drainages.

### Environmental Gradients

Principal component analysis (PCA) performed on large-scale regional variables yielded two components with eigenvalues >1, which accounted for 70.4% of the total variation (Table 3). Principal component 1 (regional gradient 1, R1) was positively loaded on catchment mean elevation, catchment elevation range, catchment mean slope, mean annual precipitation, mean summer precipitation, and summer ombrothermic index, and therefore described a south-north latitudinal gradient, mainly related to climate and topography, with increasing values for catchments with higher mean annual and summer precipitation, lower aridity, and both higher elevation and terrain relief. R2 showed marked negative loadings on catchment area, stream order, and mean flow accumulation, and was positively loaded with catchment shape index, therefore describing an upstream-downstream longitudinal gradient, with decreasing values for sites representing higher drainage areas and higher volumes of water discharged from the upper catchment.

**Table 3:** Loadings of regional and local variables on the first two principal components (PC) extracted by PCA and the eigenvalues and proportions of variance accounted for by each axis for the 117 sites in Portugal.

	PC_R_ axes			PC_L_ axes	
Regional variables	R1	R2	Local variables	L1	L2
Catchment area		−0.84	Elevation	0.67	
Catchment shape index		0.75	Channel slope	0.60	
Stream order		−0.80	Mean annual temperature	−0.80	
Catchment mean elevation	0.75		Conductivity	−0.72	
Catchment elevation range	0.78		Mean width		0.61
Catchment mean slope	0.77		Mean depth		0.93
Mean flow accumulation		−0.89	Maximum depth		0.84
Mean annual precipitation	0.86		Dominant substrate	0.71	
Mean summer precipitation	0.90				
Ombrothermic index	0.94				
eigenvalue	5.3	3.1	eigenvalue	3.7	2.3
variance (%)	44.2	26.2	variance (%)	24.6	15.4

Marked loadings are ≥ |0.60|.

PCA on instream local variables generated two components with eigenvalues >1, which accounted for 40.0% of the total variation. Local gradient 1 (L1) also represented a latitudinal variation, and was positively related to elevation, channel slope, and dominant substrate, and negatively related to mean annual temperature and conductivity, representing a shift from southern low-gradient and warmer stream reaches, with finer substrates and more productive waters, to northern higher gradient and cooler reaches, with coarser substrata and lower conductivity. L2 exhibited a habitat-size longitudinal gradient, and was positively loaded on mean width, mean depth and maximum depth, with increasing values for larger and deeper downstream sites.

### Model Averaging

**Figure 2:**
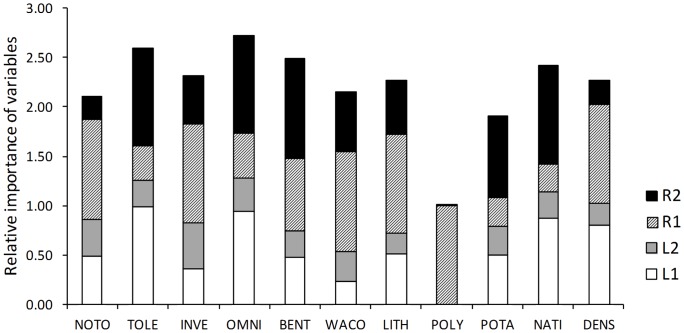
Relative importance of each variable given by the probability of the variable to be included in the best models as measured by the Akaike weights. The relative importance of each variable corresponds to the sum of its weight with the weight of its quadratic term with a maximum value of 1. Metrics abbreviations as follows: NOTO - density of non-tolerant individuals, TOLE - density of tolerant individuals, INVE - density of invertivores individuals, OMNI - density of omnivorous individuals, BENT - density of benthic individuals, WACO - density of water column individuals, LITH - density of lithophilic individuals, POLY - density of polyphilic individuals, POTA - density of potamodromous individuals, NATI - number of native species, DENS - total density of native individuals.

For the metrics with significant estimates of GLM coefficients, the number of models with substantial support (i.e. with Δi ≤2) ranged from 1 (POLY) to 7 (INVE), with cumulative Akaike weights varying from 0.49 (DENS) to 0.99 (POLY), and variability explained by the top-ranked models ranging from 21% (OMNI) to 56% (NATI) (Table 4). All coefficients of models for POTA showed no statistical support, with 95% confidence intervals overlapping zero. For all the analyzed metrics, models including regional environmental variables (R1 and R2) were more likely to be selected as more informative (Figure 2). Furthermore, regional variables showed higher support for all metrics following calculation of unconditional 95% confidence intervals (Table 4). Thus, R1 had a unimodal effect for the five metrics where it showed a larger support and higher relative importance (NOTO, INVE, WACO, LITH, and DENS), while R2 displayed a unimodal effect for NATI, and had a negative linear response for TOLE, OMNI, and BENT. On the other hand, L1 exhibited a large support and a substantial relative importance for the metrics NATI, TOLE, and OMNI, where it showed a negative linear effect. L2 displayed the weakest support for all metrics according to the 95% confidence intervals and had the lowest relative importance.

**Table 4:** Summary results of model selection for the relations between fish metrics and environmental gradients reflected in each of the two components extracted from principal component analyses of regional (R) and local (L) variables.

Metric	N	Σ*wi*	*R^2^*	R1	R1^2^	R2	R2^2^	L1	L1^2^	L2
NOTO	4	0.66	0.28	−**0.42** (−0.70, −0.15)	−**0.21** (−0.37, −0.06)			0.10 (−0.17, 0.36)		−0.05 (−0.20, 0.10)
TOLE	2	0.73	0.30	−0.14 (−0.62, 0.34)		−**0.66** (−1.11, −0.20)		−**1.52** (−2.12, −0.91)		
INVE	7	0.77	0.31	−**0.45** (−0.70, −0.20)	−**0.20** (−0.36, −0.04)	0.07 (−0.12, 0.27)		0.05 (−0.13, 0.24)		−0.07 (−0.25, 0.12)
OMNI	3	0.83	0.21	−0.17 (−0.70, 0.36)		−**0.87** (−1.34, −0.40)		−**1.01** (−1.63, −0.38)		−0.05 (−0.27, 0.16)
BENT	3	0.72	0.28	−0.42 (−1.01, 0.18)		−**0.93** (−1.31, −0.56)		−0.21 (−0.78, 0.36)		
WACO	3	0.62	0.37	−**0.46** (−0.64, −0.27)	−**0.26** (−0.41, −0.11)	0.12 (−0.09, 0.33)				−0.02 (−0.09, 0.06)
LITH	4	0.73	0.33	−**0.50** (−0.79, −0.21)	−**0.23** (−0.39, −0.08)	−0.08 (−0.27, 0.12)		0.13 (−0.18, 0.44)		
POLY	1	0.99	0.32	−2.11						
POTA	5	0.69	0.09	0.01 (−0.15, 0.16)		−0.35 (−0.72, 0.01)		0.21 (−0.25, 0.66)		0.01 (−0.06, 0.09)
NATI	2	0.66	0.56			−**0.34** (−0.48, −0.20)	−**0.08** (−0.14, −0.02)	−**0.16** (−0.26, −0.05)		0.01 (−0.04, 0.07)
DENS	4	0.49	0.36	−**0.31** (−0.43, −0.19)	−**0.16** (−0.29, −0.03)	−0.04 (−0.16, 0.08)			−0.11 (−0.23, 0.02)	

For each response variable (fish metric), the table provides the cumulative Akaike weight (Σ*wi*), calculated by summing the Akaike weights of the N best approximating models (Δi ≤2), Nagelkerke r-square of the top-ranked model (*R^2^*), and model-averaged estimates of GLM coefficients (unconditional 95% confidence intervals in parentheses) for the gradients included in the model (and quadratic term). Coefficients where the 95% confidence interval does not overlap zero are represented in bold.

### Effects of Environmental Gradients on Metrics

NOTO was primarily influenced by the regional-level gradient related to the latitudinal variation of climate and topography (R1), showing a unimodal response (Table 4). Thus, we expect density of non-tolerants to be higher in northern and central midelevation streams, with medium catchment slopes and more stable flows. The *wi* of the best models was 0.66 and the top-ranked model had a R-square of 0.28. TOLE was negatively affected by both the regional-level gradient related to the longitudinal stream continuum (R2) and the latitudinal variation of in-stream habitat conditions (L1), which means that density of tolerant fishes tend to be higher at downstream sites representing lowland streams with gentle slopes, highly productive and warm waters, and finer substrates. The best models had a *wi* of 0.73 and the R-square of the top-ranked model was 0.30.

INVE was primarily influenced by R1, showing a unimodal response to this gradient. The *wi* of the best models was 0.77 and the top-ranked model had a R-square of 0.31. OMNI, on the other hand, was negatively affected by both R2 and L1. The best models had a *wi* of 0.83 and the R-square of the top-ranked model was 0.21.

BENT displayed a negative response to R2 and, hence, tends to be positively correlated with downstream reaches. The *wi* of the best models was 0.72 and the top-ranked model had a R-square of 0.28. Conversely, WACO was primarily influenced by R1, showing a unimodal relationship along this gradient. The best models had a *wi* of 0.62 and the R-square of the top-ranked model was 0.37.

LITH also exhibited a unimodal response to R1. The *wi* of the best models was 0.73 and the top-ranked model had a R-square of 0.33. POLY was negatively responsive to R1, although the response showed poor statistical support (only one model). The cumulative *wi* was near 1 and the R-square of the model was 0.32.

NATI was primarily influenced by R2, with a unimodal response, and by L1, with a linear negative response, meaning that a maximum richness is attained in middle-order reaches with low levels of altitude, slope, and substrate, and with warm and productive waters. The *wi* of the best models was 0.66 and the top-ranked model had a R-square of 0.56. DENS displayed a unimodal response to R1. The best models had a *wi* of 0.49 and the R-square of the top-ranked model was 0.36.

## Discussion

Our study revealed that generic patterns of the fish life-history traits in Iberian least disturbed streams were primarily associated with regional features, such as catchment elevation and slope, rainfall (and, consequently, run-off), and drainage area. Over thirty years ago, the importance of landscape-stream interactions was clearly stated by Hynes [52] ‘in every respect the valley rules the stream’. The regional patterns operate on large temporal and spatial scales constraining the dynamics of smaller units, and, consequently, the resulting physical patterns will strongly influence the biological assemblages [53]–[55]. In fact, although stream fish assemblages are influenced by factors that occur at multiple scales, large scale measurements may be the best predictors of assemblage structure in studies like ours dealing with broad geographical areas [56]. Consequently, our prediction, that functional organization of fish assemblages would be associated with broad latitudinal and longitudinal gradients, was supported by our findings. Most guild metrics were related to strong north-south gradients (R1 or L1), corresponding partly to changes in climate and topography, with precipitation, altitude and slope increasing, and temperature and aridity decreasing, from south to north. This division broadly corresponds to different forms of ecosystem functioning, ranging from the colder, steeper and higher-altitude northern streams to the warmwater, and lowland southern systems. Thus, given the large geographical extent of the present study, the pronounced latitudinal gradient in fish assemblage variation was to be expected. Ferreira et al. [27] suggested a similar organisation of fluvial systems in Portugal but their work has been hampered by lack of representative network of reference sites. On the other hand, the upstream-downstream gradient (R2) also greatly influenced local assemblages at the basin level. In fact, interactions between the catchment landscapes and aquatic organisms can change predictability with longitudinal position [56]. Although studies dealing with longitudinal variation of fish assemblages in Iberian streams [57]–[59] are limited, and do not only consider least disturbed reaches, they also provided evidences of assemblage changes along these lotic systems.

We found that the density-based metrics strongly influenced by the regional gradient R1 displayed unimodal relationships, indicating lower fish densities in the northern reaches that had higher catchment relief and slope, and strong runoff events, and higher densities at some intermediate levels. This result is concordant with the findings of Oliveira et al. [44] in the least disturbed and forested areas of northern and central Portugal, who found more and larger individuals in midelevation streams with gentler slopes. Possibly, lower gradient sites, with intermediate flow levels, tend to show higher diversity of available habitats for fish. Contrariwise, in the mountainous upper catchments the environmental conditions can be restrictive, corresponding to smaller, cooler and fast-flowing habitats that could limit both fish abundance and richness [60]–[61]. Additionally, it is known that altitude influences species occurrence through water temperature [62]. For example, as long as temperatures remain cold enough for trout reproduction and growth, increased temperatures that also increase metabolism and food supply are likely to increase trout production [63]–[64]. Lower productive waters, that usually characterize the upper reaches, also have lesser abundance and diversity of invertebrate fauna [65]–[66], constraining the food supply of invertivores species. On the other hand, and given the unimodal responses of these metrics, they also decreased at the extreme opposite of the R1 gradient, i.e., in the southern stream reaches with lower catchment relief and slope, and more hydrological variability (as discussed below).

We also found a strong unimodal response for the longitudinal component of the metric ‘number of native species’. One of the most cited riverine ecosystem theories, the river continuum concept (RCC) [67], explicitly predicts changes in fish assemblages along the longitudinal abiotic gradients that occur in the river systems. Although other river ecology concepts have been developed over the last three decades, the RCC still adequately describes river zones, as the ones dealt with in this work, where longitudinal processes predominate [68]. In fact, this concept still offers stream ecologists a powerful integrative framework for research (e.g., [25], [31], [69]). In our study the upper stream reaches were characterized by *S. trutta* alone or in association with a few reophilic species. A decrease in the number of species towards headwater streams is a general pattern of the river continuum hypothesis, and as we pointed out can be largely explained by the cold temperatures and the high channel slopes that filter out other species [70]–[71]. Contrary to the typical increase in species with stream size (e.g., [25], [32]–[33], [72]–[74]), we did not find higher species richness in the larger streams of our study (20–30 m). In fact, our results seems to agree once more with the RCC which predicts maximum biotic diversity in midsize streams in response to habitat diversity and the number of niches available to different autecological types [67]. A parabolic relationship between species richness and stream order in near-natural systems was also found by Aarts and Nienhuis [31] in France. Anthropogenic disturbances have impacted streams worldwide and this can explain divergences from the predictions of the RCC, which was developed for unperturbed fluvial systems. However, even in our study, which only considered reference stream reaches, the non-linear response of species richness with measures of stream size should be interpreted in the light of the contemporary fish assemblages, because of the river impoundment that has taken place in Portugal in the last sixty years. In particular, diadromous migrations have been obstructed by large dams in some river basins, and thus relatively more diverse fish assemblages were likely to occur in the past. Independently of stream size, local variables (L1) also explained an important component of variation in species richness, suggesting that processes at the reach scale may contribute to spatial heterogeneity within the stream continuum. In fact, although the RCC has been an effective framework for understanding the structure and function of assemblages along river systems, longitudinal relationships may be clouded by local factors [75]. For example, McNeely [76] have reported an unusual pattern in species diversity along a pristine stream, with habitat diversity overriding the typical upstream-downstream gradients.

At the broadest scale, climate and topography strongly contribute to the general hydrological characteristics, temperature regime, substrate type and local slope [77]. This is particularly evident in a significant area of the northern region, where the topography and climate described earlier support permanent and coarse-bottomed streams, with low-conductive waters and dense riparian vegetation. Particularly, relief makes a major contribution to the erosive force acting on streambed, and substrate size tends to be generally larger in the steeper gradients [53]. Changes in substrates influence the dominant reproduction guild [60], and thus higher density of lithophils was generally associated with the northern and central midelevation streams. Conversely, and despite the poor statistical performance of the model, polyphilics appear to be associated with the southern areas having lower catchment relief and slope (and, consequently, finer substrates), and lower annual and summer rainfall. Since this guild is largely represented by *C. paludica*, we may conclude that this species exhibited spatial patterns that are consistent with other studies in Mediterranean streams [12], [78]. In the northern region, streams generally maintain more stable flows during periods of low rainfall, comparatively to the southern intermittent streams. Such more stable hydrological conditions would favour species having narrower water and habitat flexibility [79], and thus higher density of non-tolerants was expected. We also found higher densities of invertivores in these northern and central streams densely shaded by deciduous vegetation. These systems receive large amounts of allochthonous inputs of food provided by the riparian vegetation [80]–[82], and thus fish assemblages may include high densities of invertivores species [67]. In particular, terrestrial invertebrates can comprise more than 50% of energy intake by stream fishes and are often a preferred prey of juvenile salmonids [82]. Like the typically carnivorous *S. trutta*, the endemic invertivores cyprinids have a mouth in a superior position, allowing them to feed more effectively on terrestrial invertebrates, and thus these prey may significantly contribute to their diet [83]–[84]. Moreover, alders – the dominant riparian trees in the northern region – provide much larger inputs of invertebrates to streams than southern dominant trees as ashes or oleanders [80]. Since most of our water column species are active swimmers that feed on drifting and surface prey, we expected that this guild also had a similar association with the latitudinal gradient R1. However, these conclusions should be tested in the more general context of the links between the carbon fluxes in the Iberian stream ecosystems and the functional structure of fish assemblages [85].

Our results show strong longitudinal variability for some guild metrics, such as increasing of omnivorous, tolerant and benthic individuals with stream size. The RCC suggests that in the downstream reaches the lower trophic contribution of the riparian vegetation and the higher quantities of particulate organic matter, tend to increase the proportion of generalist feeders [86]. This prediction was also confirmed for European streams [14], [29], [87], and for fish assemblages of four continents [25]. We expected a similar response for the density of tolerants, since both metrics are strongly associated in Mediterranean streams, increasing along the longitudinal gradient [14]. Additionally, the present study also provides support that local habitat conditions (i.e. gradient L1) may be of great importance in determining the density of omnivorous and tolerant individuals. In fact, for downstream sites with similar drainage areas, we found higher proportion of these two guilds in the southern stream reaches with gentler slopes, warmer temperatures, finer substrates and higher conductivities. But levels of sedimentation also increase with organic matter (even in low disturbed rivers), and thus the density of species which are adapted to substrate may increase along the upstream-downstream gradient, contrary to the ones that feed on invertebrates in the water column. Reinforcing this hypothesis is the fact that our dominant benthic species (endemic cyprinids) have mouths in a inferior position that allow them to feed very effectively on the riverbed [83].

A valid understanding of patterns and processes in ‘natural’ conditions is an essential prerequisite for viable ecosystem management [88]. The findings of our study emphasized the need to use a multi-scale approach to fully assess the factors that govern the functional organization of biotic assemblages in reference conditions, as well as to improve biomonitoring and restoration of fluvial ecosystems. The hierarchical models postulate that the units of streams are constrained by larger scales, from biota to local habitat to basin. To identify the spatial scale that explain most of the variation in biotic composition is to find the scale at which the most important physical/chemical processes constraining the assemblages can be found [89]. Thus, the effectiveness of actions that strive to improve river health rely on the ability to distinguish between and understand the multiscale environmental gradients. In spite of these perspectives, most stream restoration efforts often concentrate on a discrete river reach or segment involving localized interventions even when larger-scale processes may be paramount [90]–[91]. However, the results of our study should be viewed with caution, as they represent an ecological snapshot. Temporal variation, which could not be detected by our single sampling at each site, may play a role in understanding the spatial distribution of fish guilds. Future studies should consider monitoring fish assemblages at appropriate temporal scales, to provide additional insights for understanding the environmental correlates of the functional structure of Iberian fish assemblages and their implication for river management.

## Supporting Information

Table S1 Criteria for scoring qualitative variables related to human disturbance. Variables were scored to the degree they deviated from minimally disturbed conditions (from 1 for no deviation, to 5 for highly degraded).
(DOCX)
