# Supplementary material for: Modelling Stream-Fish Functional Traits in Reference Conditions: Regional and Local Environmental Correlates
Source: PLoS One. 2012 Sep 24;7(9):e45787. doi: 10.1371/journal.pone.0045787 (PMC3454361; doi:10.1371/journal.pone.0045787)
Supplement: Table S1 — Criteria for scoring qualitative variables related to human disturbance. Variables were scored to the degree they deviated from minimally disturbed conditions (from 1 for no deviation, to 5 for highly degraded). (DOCX) [file pone.0045787.s001.docx]

**Table S1. Criteria for scoring qualitative variables related to human disturbance.** Variables were scored to the degree they deviated from minimally disturbed conditions (from 1 for no deviation, to 5 for highly degraded).

| Variables | Criteria |
| --- | --- |
| Catchment |  |
| Agriculture | < 10% agriculture and < 3% intensive farming (1) to > 50% agriculture or > 10% intensive farming (5) |
| Urbanization | < 1% (1) to > 25% (5) urban |
| Forest and semi-natural | > 90% (1) to < 30% (5) natural land uses |
| *Eucalyptus* sp. monoculture | < 10% (1) to > 70% (5) *Eucalyptus* sp. monoculture |
| Burned area | < 10% (1) to > 70% (5) burned drainage area at least from five years ago |
| Segment |  |
| Land use | < 10% nonnatural (1) to > 40% intensively cultivated land/intensive silviculture (5) |
| Urbanization | < 1% (1) to > 25% (5) urban |
| Riparian disturbance | no or minor impacts (1) to complete riparian vegetation removal (5) |
| Morphological alteration | negligible (1) to complete channelization and bank hardening (5) |
| Sediment load | < 5% of the bottom with fine sediments, and little turbidity (1) to > 75% of the bottom with fine sediments, or high turbidity (5) |
| Reach |  |
| Alien fish abundance | no (1) to > 50% (5) aliens |
| Hydrological disturbance (dams upstream) | little (1) to extreme (5) deviation from the natural annual flow regime |
| Probability of invasion by alien lentic species (dams downstream) | very low (1) to high probability (5) of invasion by alien lentic species, as a function of the proximity and size of hydroelectric power plants downstream from the site |
| Nutrient & organic contamination | unpolluted (1) to extremely polluted (5) |
| Oxygen concentration | no (1) to extreme (5) deviation from the natural seasonal variation |
